# Muscle co-activity tuning in Parkinsonian hand movement: disease-specific changes at behavioral and cerebral level

**DOI:** 10.3389/fnhum.2015.00437

**Published:** 2015-08-05

**Authors:** A. M. M. van der Stouwe, C. M. Toxopeus, B. M. de Jong, P. Yavuz, G. Valsan, B. A. Conway, K. L. Leenders, N. M. Maurits

**Affiliations:** ^1^Department of Neurology, University Medical Center Groningen, University of GroningenGroningen, Netherlands; ^2^Biomedical Engineering, University of StrathclydeGlasgow, UK

**Keywords:** Parkinson's disease, step-tracking, fMRI, EMG, kinematic parameters, muscle co-activity

## Abstract

We investigated simple directional hand movements based on different degrees of muscle co-activity, at behavioral and cerebral level in healthy subjects and Parkinson's disease (PD) patients. We compared “singular” movements, dominated by the activity of one agonist muscle, to “composite” movements, requiring conjoint activity of multiple muscles, in a center-out (right hand) step-tracking task. Behavioral parameters were obtained by EMG and kinematic recordings. fMRI was used to investigate differences in underlying brain activations between PD patients (*N* = 12) and healthy (age-matched) subjects (*N* = 18). In healthy subjects, composite movements recruited the striatum and cortical areas comprising bilaterally the supplementary motor area and premotor cortex, contralateral medial prefrontal cortex, primary motor cortex, primary visual cortex, and ipsilateral superior parietal cortex. Contrarily, the ipsilateral cerebellum was more involved in singular movements. This striking dichotomy between striatal and cortical recruitment vs. cerebellar involvement was considered to reflect the complementary roles of these areas in motor control, in which the basal ganglia are involved in movement selection and the cerebellum in movement optimization. Compared to healthy subjects, PD patients showed decreased activation of the striatum and cortical areas in composite movement, while performing worse at behavioral level. This implies that PD patients are especially impaired on tasks requiring highly tuned muscle co-activity. Singular movement, on the other hand, was characterized by a combination of increased activation of the ipsilateral parietal cortex and left cerebellum. As singular movement performance was only slightly compromised, we interpret this as a reflection of increased visuospatial processing, possibly as a compensational mechanism.

## Introduction

The direction of voluntary hand movement along the wrist originates from cerebrally encoded vectors, without a direct link to specific muscles to effectuate their contraction. Regarding the effector system, however, movement in some directions is dominated by activity of only one agonist muscle, while other directions require coordinated simultaneous activation, or, “co-activation” of multiple agonists. This directional tuning highlights the highly adaptable outflow structure of motor commands that underpin goal directed movements. The basal ganglia (BG) are known to modify the cortically generated motor plan by selecting appropriate muscles and inhibiting undesired motor activity (Alexander et al., 1986; Mink, 1996; Middleton and Strick, 2000; Rubchinsky et al., 2003; de Jong and Paans, 2007).

In studies addressing these aspects of direction tuning in motor control, center-out step-tracking tasks (Hoffman and Strick, 1999) are commonly used. In such tasks, subjects are required to make hand excursions into various directions. By combining this motor paradigm with functional brain imaging, we aimed to demonstrate that the BG play an important role in the organization of tuned muscle co-activity. We hypothesized that increased BG activity would be found in movement excursions requiring multiple muscles to be simultaneously active (co-active), which we defined as “composite movements,” to be distinguished from “singular movements” requiring the activity of one dominant agonist. Given the association between pathophysiological BG changes and characteristic movement impairments in Parkinson's disease (PD) (DeLong and Wichmann, 2009), we included PD patients, expecting to find reduced BG activity during movements requiring highly tuned muscle co-activity, when compared to healthy subjects. This concept finds support from the observation that muscle tuning is indeed impaired in PD as patients show insufficient inhibition of antagonist muscles, which causes co-contraction of agonist and antagonist muscles (Meunier et al., 2000). To gain insight in the impaired selection of highly tuned muscle co-activity in PD patients, the here employed center-out step-tracking task thus enabled the comparison of movements executed with different degrees of muscle tuning between PD patients and healthy subjects both at behavioral level, using kinematic and electromyography (EMG) parameters, and at the cerebral level by using functional magnetic resonance imaging (fMRI).

The center-out step-tracking task employed a manipulandum that enabled measurement of hand movement along the wrist, made toward eight different targets. A priori, we made a distinction between movement directions requiring either more or less muscle co-activity based on what is known from previous work on step-tracking (Hoffman and Strick, 1999). Therefore, the first step of the present study was to validate the distinction between composite and singular movements in healthy subjects. In addition to the EMG data, we analyzed kinematic parameters to confirm that a pattern of composite muscle activity indeed results in a movement profile that differs from a singular muscle activity pattern. Finally, differences in brain activation patterns related to these tasks were assessed with fMRI using an event-related design. This entails, that we contrasted composite and singular movements to identify differences between PD patients and healthy subjects regarding the cerebral organization of movement with different degrees of muscle tuning. We expected to find impaired performance by PD patients, reflected in increased reaction times and more extensive muscle co-activity, while at the cerebral level, we hypothesized to find reduced brain activation in specifically the BG and interconnected circuitry. To our knowledge, this study is the first to use a center-out step-tracking task for the purpose of investigating muscle tuning organization on an output level (EMG and kinematics) as well as on brain (organizational) level in PD patients as well as in healthy subjects.

## Methods

### Subjects

The study was approved by the Medical Ethical Committee of the University Medical Center Groningen (UMCG). Patients were recruited at the outpatient clinic for movement disorders at the UMCG and healthy subjects were recruited by advertisements in local newspapers. Subjects participated after full explanation of the study's purpose, protocol and risks, and provided informed consent in accordance with the Declaration of Helsinki (2008). All subjects participated in two experimental sessions, the second of which included fMRI. Twelve patients with idiopathic PD experiencing mild to moderate clinical symptoms were recruited. Patients were assessed by the Unified Parkinson's Disease Rating Scale (UPDRS) (Fahn et al., 1987), and Hoehn–Yahr disability scale (Hoehn and Yahr, 1967). In addition, 18 healthy gender and age matched subjects were recruited. Patients had to have a stable response to medication, and to reduce medication effects, had to refrain from taking their morning dose of levodopa, or dopamine agonists (overnight withdrawal). All subjects had to be right handed as assessed by the Annett Handedness Scale (Annett, 1970). Exclusion criteria for both groups were a history of epileptic seizures, head injury, neurological diseases (for patients: other than PD), psychiatric diseases, or the use of any type of medication affecting the central nervous system. Also, during a brief neurological physical examination it was ensured that subjects had (corrected-to-) normal vision. Patients who could either not abstain from their levodopa use or had a Mini Mental State Examination (MMSE, Cockrell and Folstein, 1988) score <26 were excluded. Patients with Parkinsonism other than PD or the tremor-dominant type of PD, which might be regarded as a PD subtype (Josephs et al., 2006), were also excluded to obtain a maximally homogeneous group. Subjects came in for the behavioral and fMRI experiments on two separate days, with a maximum interval of two weeks. During the first visit, subjects performed the task in sitting position and additionally practiced one block of the task in the supine position inside a dummy MR scanner. During the second visit subjects practiced task performance prior to fMRI data collection, again for one block.

### Experimental set-up

**Figure 1:**
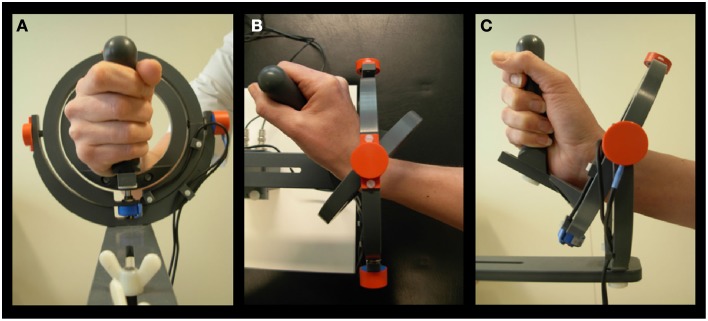
**Manipulandum. (A)** Hand held in neutral position. **(B)** Movement in 0° direction, corresponding to extension. **(C)** Movement in 90° direction, corresponding to radial deviation (Toxopeus et al., 2012).

All subjects performed a visual step-tracking task with the right hand, using a magnetic resonance (MR) compatible manipulandum (Figure 1). The applied manipulandum is a joystick-like device that can rotate in two perpendicular planes allowing all combinations of wrist flexion-extension and wrist ulnar-radial deviation. Subjects were comfortably positioned with the right arm supported by an armrest. The hand was positioned in a vertical plane and subjects grasped the manipulandum handle. The right wrist joint was positioned in the center of the two concentric rings composing the device. The fingers were taped to the thumb reminding subjects to hold the grip with all fingers. Prior to the start of each block of step-track movements subjects were requested to hold their wrist in a neutral position, i.e., in the center of the manipulandum, and the center of the screen was adjusted to the position of the cursor corresponding to this neutral position (center point on screen). This was done to make sure that anatomic variation of hands did not interfere with task execution. The range of wrist movement from this position was checked to ensure that subjects were able to move freely in each direction. To provide visual feedback on task performance, angular displacement was measured in both (X and Y) planes by potentiometers mounted in-line with the axes of the manipulandum rings and displayed as a cursor (a 5 × 5 mm closed square) following digitization using a Power 1401 analog-to-digital converter controlled using Spike 2 [Cambridge Electronic Design (CED), Cambridge, UK].

To investigate kinematic and EMG results for all movements, data of the behavioral experiment were used. Pilot experiments comparing performance of the step-tracking task in sitting and supine positions had shown that there were no differences in kinematic and EMG data between the two positions and the EMG data from the behavioral experiment are not distorted by fMRI-related artifacts. During scanning, subject performance was visually monitored by a second computer in the MR control room.

### Task

**Figure 2:**
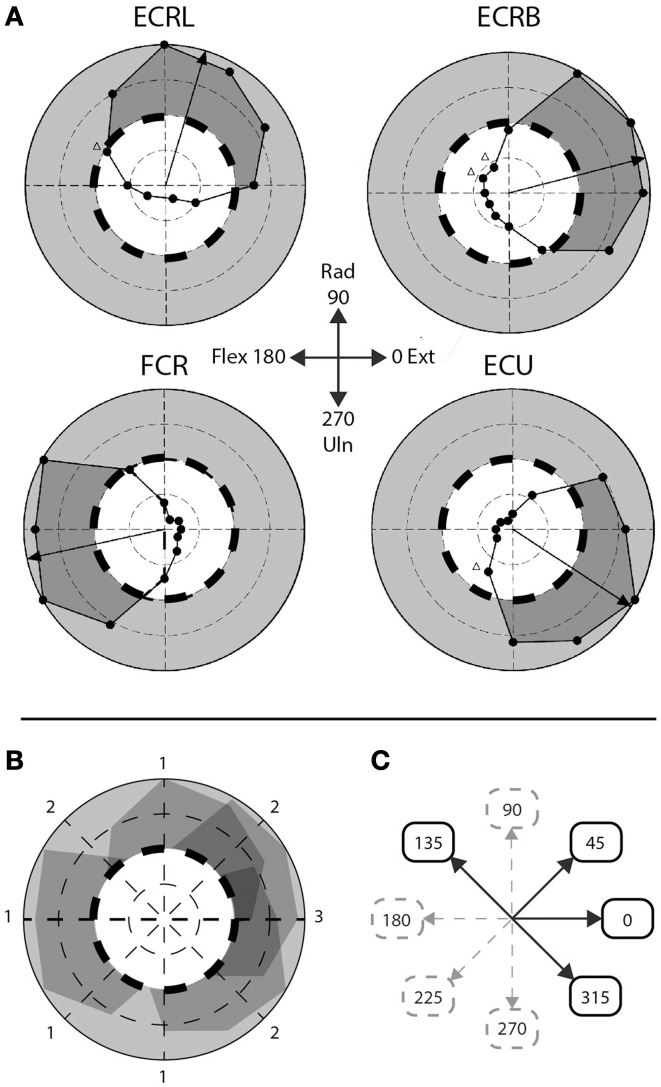
**Step-tracking task: composite vs. singular movement directions**. **(A)** Figure adapted from Hofmann and Strick. Scaled EMG activity; dashed line indicates 50% scaled EMG activity, gray area indicates >50% scaled EMG activity, dark gray area indicates subject's performance >50% EMG activity. ECRL, m. extensor carpi radialis longus; ECRB, m. extensor carpi radialis brevis; FCR, m. flexor carpi radialis; ECU, m. extensor carpi ulnaris. **(B)** Overlay of the four muscles depicted in **(A)**. Numbers indicate number of muscles >50% EMG activity for that particular movement direction. **(C)** A priori division of composite and singular movements based on **(B)**. Composite movements are depicted as black arrows, singular movements as gray, dashed arrows.

Subjects were asked to place their cursor in the “center box” (3 × 1.5 cm open rectangle). A warning cross preceding the appearance of the target was displayed in this center box for 1 s. After disappearance of the warning cross, a target stimulus (3 × 1.5 cm open rectangle) appeared at one of eight possible positions (Figure 2C). The time intervals between warning cross and target were randomized (jitter: 0.8 ± 0.4 s). All eight directional stimuli had the same distance relative to the center (20°) of the screen and were equally spaced. Regarding the hand position in the manipulandum, movements in 0° and 180° directions corresponded with extension (right) and flexion (left), respectively, whereas movements in 90° and 270° directions corresponded with radial (up) and ulnar (down) deviation, respectively.

After moving toward the target, subjects were required to hold the cursor in the target box until the target box disappeared (3 s after appearance of the target stimulus) whereupon they return to the neutral (center) position. Each of such movement trials, which was coined a full step-track movement, lasted 5 s. After every 10 step-tracks, there was a short break of 4 s. One step-track block consisted of 40 stimuli, five for each of the each different directions presented in fixed randomized order (randomized but in the same order for every subject). The entire task consisted of four blocks.

### A priori division into composite and singular movement

To discriminate between movements that require a higher level of muscle co-activity and movements requiring less muscle co-activity, the eight movement directions were a priori divided in two groups, based on the step-tracking study of Hoffman and Strick (1999). We used a profile of scaled EMG activity of the same four arm muscles we investigated as a template to determine the number of muscles actively contributing to movement for each direction (Figures 2A,B). Muscles were regarded to significantly contribute to distinct movement directions when activity was larger than 50% of the maximum scaled EMG activity over all directions for that muscle; meaning that at least half of the muscle's peak level of agonist burst activity was required for movement in that particular direction. Since Hoffman and Strick investigated 12 directions, the number of muscles contributing to the four movement directions in our study (45°, 135°, 225°, and 315°) were determined by averaging the number of muscles contributing to movement in their directions 1 and 2, 10 and 11, 7 and 8, and 4 and 5 o'clock, respectively. Movement directions involving multiple (>1) lower-arm muscles were regarded as requiring highly tuned muscle coordination during the initial agonist burst and were labeled “composite movements.” The second group of movement directions, dominated by activity in single agonist muscles, was labeled “singular movements.” This resulted in a balanced decomposition into two groups of four movement directions each; a “composite” group with movements directed in 0°, 45°, 135° and 315°, and a “singular” group consisting of movement directions 90°, 180°, 225°, and 270° (Figure 2C). Note that for example movement to direction 0° (full wrist extension) is considered a composite movement, since accomplishing movement in this direction requires co-activity of multiple extensor muscles (Hoffman and Strick, 1999). Realizing that this a priori division in singular and composite movements is based on a single human dataset, we later validated this division using our own EMG data in healthy subjects (see Section EMG Data).

### EMG data recording

To record muscle activity, four bipolar Ag/AgCl surface electrode pairs were placed on the lower (right) arm muscles m. Extensor carpi radialis longus (m. ECRL), m. Extensor carpi radialis brevis (m. ECRB), m. Flexor carpi radialis (m. FCR), and m. Extensor carpi ulnaris (m. ECU). A reference electrode was placed on the dorsal side of the left hand. To improve skin conductance, the skin was pre-treated with a scrub gel and subsequently a conductive paste was applied. EMG electrodes were placed longitudinally with respect to the muscle fibers and attached approximately 1.5 cm apart. The muscles were identified by palpation, using maximum voluntary contractions (EMG) toward the specific pulling direction of each individual muscle. To diminish cross-talk, we verified that movement toward the pulling direction mainly elicited activity in the EMG channel belonging to that specific muscle. EMG data were recorded at 5000 Hz using Brain Vision Recorder software (Brain Products GmbH Munchen, Germany).

### MRI characteristics

fMRI data acquisition was performed using a 3 Tesla Magnetic Resonance System (Philips, Best, Netherlands) with a standard six-channel head coil. T2^*^-weighted, 3D functional images were obtained using multislice echo planar imaging (EPI) with an echo time (TE) of 30 ms and a repetition time (TR) of 2000 ms. Per TR 39 axial slices, with a field of view (FOV) of 224 mm, flip angle of 5° with a 64 × 64 matrix and isotropic voxel size of 3.5 × 3.5 × 3.5 mm were acquired. Functional scanning included 106 volumes per block. To provide anatomical information (isotropic voxel size 1 × 1 × 1 mm), additional T1-weighted 3D anatomical scans with an axial orientation and a matrix size of 256 × 256 mm were obtained.

### Analysis of kinematic and electromyography data

#### Kinematic data

Kinematic parameters for comparison of task execution between groups were derived using the X and Y displacement measured by the two potentiometers integrated in the manipulandum. The kinematic data were further analyzed using Matlab (Matlab R2007b, Mathworks, Natrick, USA). A custom-made script was used to determine a set of kinematic variables. For each individual subject reaction time (RT) and peak velocity (PV) were determined for each movement. RT was determined as the time (in ms) between stimulus presentation and movement onset. Movement onset was identified visually by a sudden change in total displacement ($\sqrt{X^{2}+Y^{2}}$) of the manipulandum. PV was determined by the maximum of the velocity, calculated as the numerical first-order derivative of the total displacement, in degrees per second. Means and standard deviations (as a measure of variability) of RT and PV per direction of movement were calculated per subject.

#### EMG data

EMG data were exported to Matlab, where they were down sampled to 100 Hz, high-pass filtered (Butterworth Zero Phase shift filter with a cut-off of 10 Hz) and full-wave rectified (Meyers et al., 2003) by using a custom made script. To enable comparison of relative EMG activity between subjects, EMG data were normalized by the maximum EMG over all experimental trials for each muscle and subject. Next, we calculated the number of muscles that significantly contributed to movement in each direction, for each of our subjects. This was primarily done to verify the a priori division into singular and composite movements (see Section Task) and, secondly, to determine differences in the number of involved muscles between groups. Muscles were regarded as significantly contributing to movement in a specific direction when reaching a cut-off value of 0.5 (scaled EMG activity). The number of active muscles, indicating the amount of muscle co-activation, was further referred to as the activity index (AI) which could range theoretically from 0 to all 4 muscles.

To quantify the extent of specialized muscle activity, we calculated the differentiation quotient (DQ) by dividing the mean scaled EMG activity of the direction in which a muscle was most active by the mean scaled EMG activity of that muscle for the seven remaining directions. This was accomplished for each muscle and individual subject separately. DQ, thus, provided insight in whether a muscle was specifically active in a distinct direction, or equally active in multiple directions, i.e., a higher DQ indicated a higher extent of specialized activity for a specific muscle, whereas a lower DQ corresponded with a less specialized activity pattern of that muscle.

#### Statistics

Statistical analysis was performed using PASW 18 (SPSS, Inc., Chicago IL). First, we used the Shapiro–Wilk test of normality to check the distribution of the data. Kinematic variables that were not normally distributed and were right-skewed were transformed using a Log^10^ transformation (in case data were not normally distributed for one group, data of both groups were transformed). Separate mixed design ANOVAs were employed to assess general significant differences for all kinematic variables (PV and RT), and EMG variables AI and DQ. Before performing mixed ANOVAs, the assumption of sphericity was tested on each variable using Mauchly's test. If the assumption was rejected the Greenhouse–Geisser correction was applied. The between-subject variable for the mixed ANOVAs was “group,” (two levels: PD patients and healthy subjects). For kinematic parameters and AI, the within-subject variable was “movement direction” (two levels: “composite” and “singular” movement). For DQ, the within-subject variable was “muscle” (four levels: “m. ECU,” “m. ECRL,” “m. ECRB,” and “m. FCR”). Main effect of muscle was further investigated employing Bonferroni corrected pairwise comparisons. The significance level was set at α = 0.05.

### fMRI data analysis

Processing of images and statistical analyses were conducted using Statistical Parametric Mapping (SPM) version 5 (2005, Wellcome Department of Cognitive Neurology, London, UK; http://www.fil.ion.ucl.ac.uk/spm). Pre-processing included standard slice time correction, realignment and co-registration of functional and anatomical scans. Images were normalized to the template of the Montreal Neurological Institute (MNI). Next, images were smoothed using a Gaussian filter of 8 mm full width at half maximum (FWHM). Event related analysis was performed; events were defined as the appearance of peripheral target stimuli in the step-tracking task. Brain activations were computed according to the standard statistical procedures in SPM. Statistical parametric maps per subject (first level analysis) were derived using a linear multiple regression model with event-related regressors and movement parameters as regressors of no interest to account for head movement-related effects. Scans were checked for head motion; all scans had maximally 3 mm translational motion and maximally 0.08° rotational motion. Two comparisons (T-contrasts) between the two types of directions (Composite > Singular and Singular > Composite) were generated at first level. The activation maps of the two between-task comparisons at first level were entered in separate ANOVAs (flexible factorial design) for initial statistical analysis of differences within the group of healthy subjects. These first level results were further used for statistical analysis of differences between groups at second level. To enable comparison of task-related differences between patients and healthy subjects, we used exclusive masking with a threshold of *p* = 0.05. Note that exclusive masks remove all voxels reaching significance in one contrast that overlap with the significant voxels in the other contrast, thereby enabling direct comparison of differences in activation patterns between healthy subjects and patients.

We primarily looked for effects in the BG/thalamus, premotor cortex (PMC), supplementary motor area (SMA), parietal cortex and cerebellum. Previous studies indicated that these specific areas are subject to changes related to PD (Playford et al., 1992; Samuel et al., 1997; Sabatini et al., 2000; Yu et al., 2007; Ma et al., 2009). We therefore determined a restricted volume including the BG and thalamus for statistical analysis with a small volume correction. This small volume was obtained by using a spherical volume of interest (VOI) [radius of 30 mm (15 voxels)] with a center placed at coordinate [0, 0, 0]. For changes of activation in the areas of interest, we used a threshold for voxel response height of *p* = 0.01 (cluster uncorrected and extent threshold of *k* = 30 voxels). To identify effects in cortical areas as well as in the cerebellum, voxel values were thresholded at a voxel response height of a liberal *p* = 0.01 (uncorrected) with an extent threshold of *k* = 10 voxels.

Activations in other regions were reported only when *p* < 0.001 (uncorrected, extended voxel threshold of *k* = 10 voxels). Activated brain regions were identified by rendering group activation maps onto the Automated Anatomical Labeling (AAL) template and Brodmann template in MRICron (Rorden et al., 2007).

## Results

### Subjects

Twelve PD patients [mean age: 59 ± 9 (range 38–69)] and 18 healthy subjects [mean age: 59 ± 5 (range 51–69)] participated in this study. One healthy subject was excluded from the behavioral part of the study, due to a technical problem that occurred while recording the kinematic data. An independent samples *T*-test revealed there were no significant age differences between patients and healthy subjects (*p* = 1.000). The clinical characteristics of the youngest patient were similar to those of the older patients. Moreover, this patient was not known to have genetic mutations and was therefore included despite her young age. A Mann–Whitney *U*-test showed that the gender distribution was similar between groups [7/12 male (PD), 9/18 male (controls), *p* = 0.723]. Similar testing ascertained that MMSE scores were also comparable between groups; the median MMSE score was 29 for PD patients, and 29 for healthy subjects (*p* = 0.113). The symptomatic state of all patients was described by their UPDRS and Hoehn and Yahr scores (see Table 1). Regarding the laterality of rigidity in PD patients, in 8/12 patients the right arm was more affected (difference in UPDRS of 1 point), in 2/12 patients the left arm was more affected (difference in UPDRS of 1 point). In 2/12 patients severity of rigidity did not differ between arms.

**Table 1:** **Clinical characteristics**.

**Patient number**	**Age**	**Sex**	**MMSE**	**UPDRS**	**H&Y**	**Lat. Rigidity**	**LLED**
1	69	M	29	36	3	R	1560
2	57	F	29	15	2	R	1045
3	48	F	28	18	1.5	L	440
4	60	M	28	12	1.5	L	132
5	60	M	29	18	1.5	R	180
6	64	M	29	23	1.5	R	714
7	69	M	27	26	2	R = L	800
8	54	M	28	26	1.5	R	600
9	60	F	29	27	1.5	R	615
10	62	F	28	18	2	R	540
11	63	M	28	25	2	R	537
12	38	F	29	14	2.5	R = L	600

MMSE, Mini Mental State Examination; UPDRS, Unified Parkinson's Disease Rating Scale; H&Y, Hoehn and Yahr disease stage; Lat. Rigidity, laterality of rigidity, e.g., left or right arm. LEDD, levodopa equivalent daily dosis = levodopa dose (mg) + (0.3 ^*^ levodopa dose if using entacapone with each dose) + (slow release levodopa ^*^ 0.7) + (bromocriptine ^*^ 10) + (ropinirole ^*^ 20) + (pergolide ^*^ 100) + (pramipexole ^*^ 100) + (apomorphine ^*^ 10) (Esselink et al., 2004).

### Kinematic results

#### Reaction time

Regarding median reaction time, we found an ordinal interaction effect between group and movement direction [*F*_(1, 33)_ = 5.189, *p* = 0.029], which indicated that the difference in RT between composite and singular movements was larger in PD patients than in healthy subjects. There was no interaction effect regarding RT variability. Overall, composite movements required longer RTs [main movement direction effect; *F*_(1, 33)_ = 32.126, *p* < 0.001] and resulted in higher RT variability [main movement direction effect; *F*_(1, 33)_ = 9.466, *p* = 0.004]. Moreover, PD patients showed longer RTs [main group effect; *F*_(1, 33)_ = 8.290, *p* = 0.007] and higher RT variability [main group effect; *F*_(1, 33)_ = 10.467, *p* = 0.003].

#### Peak velocity

An ordinal interaction effect between group and movement direction [*F*_(1, 33)_ = 3.310, *p* = 0.026, see Table 2] indicated that the difference in peak velocity between composite and singular movements was larger in PD patients in comparison to healthy subjects. A main direction effect was found, which implied that composite movements were performed with higher PV [*F*_(1, 33)_ = 3.498, *p* = 0.036]. No group effect was found.

**Table 2:** **Kinematic parameters**.

		**Healthy**	**PD**	**Interaction**	**Group**	**Direction**
		**(*n* = 17)**	**(*n* = 12)**	***p***	***p***	***p***
**RT**						
Mean	*C*	208 (43)	244 (114)^*^	0.029	0.007	<0.001
	*S*	193 (50)	223 (63)^*^			
Var.	*C*	85 (37)	116 (80)^*^	–	0.003	0.004
	*S*	74 (22)	100 (48)^*^			
**PV**						
Mean	*C*	146 (28)	138 (35)	0.026	–	0.036
	*S*	145 (22)	129 (30)			
Var.	*C*	28 (10)^*^	31 (27)	–	–	–
	*S*	27 (7)^*^	27 (28)^*^			

*Statistic results for kinematic parameters. RT, reaction time (ms); PV, peak velocity (degrees/second); Var, variability; C, composite; S, singular. Means and standard deviations are shown*.

* *Median (interquartile range)*.

### EMG parameters

#### Activity index (AI)

An ordinal interaction effect between group and movement direction was found (see Table 3), which indicated that the difference between the number of muscles involved in composite vs. singular movement was smaller in PD patients than in healthy subjects [*F*_(1, 27)_ = 10.397, *p* = 0.003]. A main movement direction effect was found: regardless of group, the (a priori defined) composite movements did indeed involve more muscles than singular movements, as indicated by AI [*F*_(1, 27)_ = 59.257, *p* < 0.001]. Moreover, a main group effect was found, revealing that PD patients showed a higher overall AI than healthy subjects [*F*_(1, 27)_ = 13.568, *p* = 0.001].

**Table 3:** **EMG parameters**.

	**Healthy**	**PD**	**Interaction**	**Group**	**Direction**
	**(*n* = 17)**	**(*n* = 12)**	***p***	***p***	***p***
**AI**					
Composite	3.3 (1.0)^*^	3.8 (0.5)^*^	0.003	0.001	<0.001
Singular	1.8 (0.9)^*^	3.1 (1.3)^*^			
**DQ**	2.0 (0.6)	1.6 (0.3)		0.028	
			**Muscle *Post-hoc***		
**DQ**			***p***	**Effect**	
m. ECU	2.7 (1.1)	2.2 (1.0)	0.001	ECU > ECRL	
			0.013	ECU > ECRB	
			<0.001	ECU > FCR	
m. ECRL	1.7 (0.7)^*^	1.3 (0.2)	0.041	ECRL > FCR	
m. ECRB	2.1 (0.7)	1.5 (0.7)	0.004	ECRB > FCR	
m. FCR	1.1 (0.5)^*^	1.1 (0.2)^*^	See above		

*Statistic results for all EMG measures. AI, activation index; DQ, differentiation quotient. Means and standard deviations are shown*.

* *Median (interquartile range)*.

*AI, activation index; DQ, differentiation quotient; m. ECU, (musculus) extensor carpi ulnaris; m. ECRL, extensor carpi radialis longus muscle; m. ECRB, extensor carpi radialis brevis muscle; m. FCR, flexor carpi radialis muscle*.

#### Differentiation quotient (DQ)

**Figure 3:**
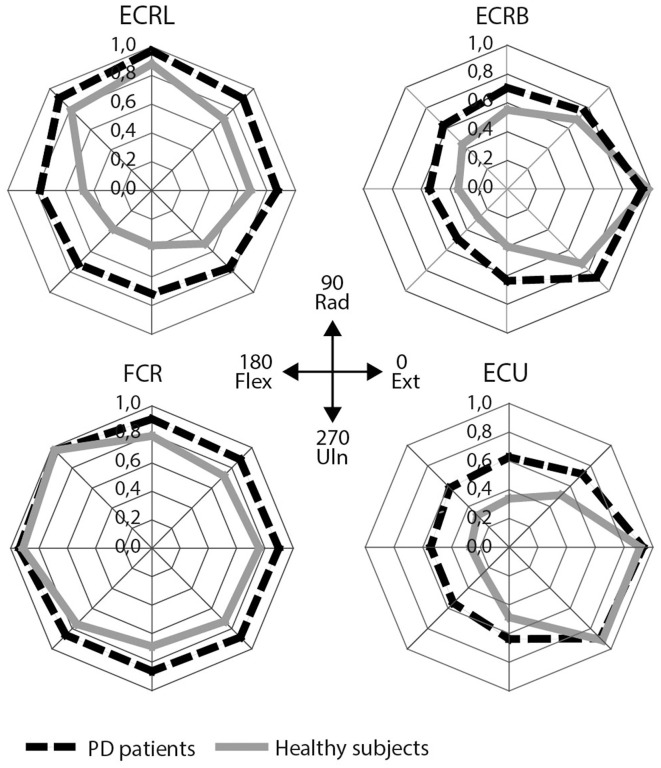
**EMG results: mean scaled EMG activity for all directions for each muscle**. Mean scaled activity is shown per group. PD patients are depicted as black, dashed lines, healthy subjects as gray lines. ECRL, m. extensor carpi radialis longus; ECRB, m. extensor carpi radialis brevis; FCR, m. flexor carpi radialis; ECU, m. extensor carpi ulnaris.

Visually, the EMG activity patterns between the two groups were clearly different (Figure 3). Healthy subjects showed more specialized EMG activity than PD patients, as reflected in each of the investigated muscles being more distinctly active in a specific direction. The muscle activity configurations representing healthy subjects were almost encapsulated in the PD patient's configurations. This observation was quantified by our measure for differentiation of muscle activity (DQ): a main group effect showed that patients had lower DQ scores than healthy subjects [*F*_(1, 25)_ = 5.394, *p* = 0.028]. Additionally, we found a main effect of muscle [*F*_(3, 25)_ = 17.048, *p* < 0.001] implying that some muscles showed a more specialized activity pattern than others. *Post-hoc* analysis revealed that m. ECU had the highest DQ compared to the other muscles, while m. FCR had the lowest. For m. ECRL and m. ECRB, DQ scores were similar (details in Table 3). No interaction effect was found.

### fMRI results

#### Within-group comparisons: Healthy subjects

**Figure 4:**
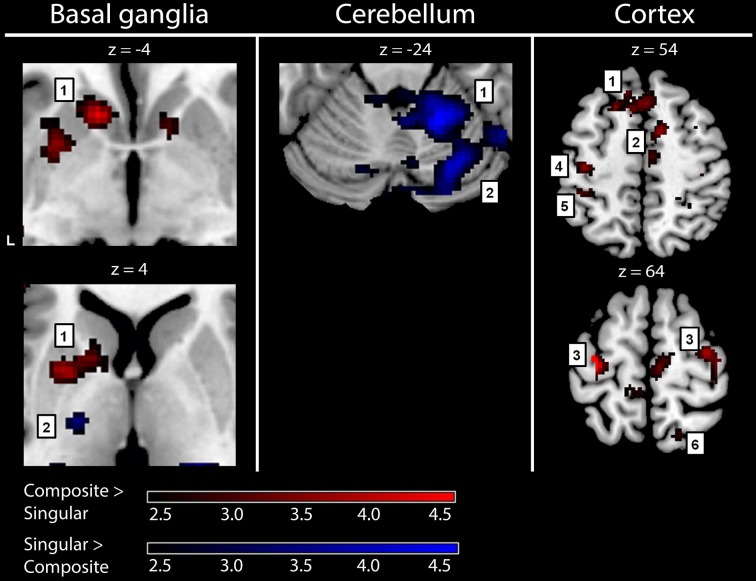
**fMRI results: activations in healthy subjects**. Red activations: composite > singular, blue activations: singular > composite. Basal ganglia: 1, left striatal cluster; 2, thalamic cluster. Cerebellum: 1, anterior (lobule IV/V) cerebellum; 2, posterior (crus 1) cerebellum. Cortex: 1, medial prefrontal (BA9); 2, supplementary motor area (BA6); 3, (dorsal) premotor cortex; 4, primary motor cortex (BA4); 5, primary sensory cortex (BA2); 6, superior parietal cortex (BA7). For visualization purposes, all activations are shown above a threshold of *Z* = 2.4 [corresponding to uncorrected voxel level *p* < 0.01, without a restriction of cluster size (k)]. The z-coordinates indicate the position of the shown transversal planes relative to the AC-PC plane. Activations were rendered on the standard anatomical (ch2) template of MRICron (Rorden et al., 2007). L, left hemisphere.

To gain optimal insight in changes in brain activation patterns in PD patients, as compared to healthy subjects, activations related to the composite and singular movement conditions were first identified in healthy subjects (Figure 4 and Table 4). We found that movement requiring more synergistic modulation (Composite > Singular) evoked a significant cluster of left striatal activation. Additionally, Composite > Singular revealed increased cortical activations comprising the SMA (BA6) and dorsolateral PMC (BA6) of both hemispheres, while contralateral to the side of movement we found increased activation in the medial prefrontal cortex (BA9), primary motor cortex (M1, BA4), and primary visual cortex (V1, BA17/18). Furthermore, the ipsilateral superior parietal cortex (BA7) showed more activation during composite movements. Healthy subject movement requiring less muscle tuning (Singular > Composite) was related to increased activations in the left (contralateral) ventral lateral thalamus and ipsilateral anterior (lobule IV/V) and posterior (crus 1) cerebellum. In addition, the right (ipsilateral) hippocampus showed increased activation related to singular movements.

**Table 4:** **fMRI results**.

	**Composite > Singular**							**Singular > Composite**						
	**HS**	**PD↓↑**	***x***	***y***	***z***	***T***	***p (uncorrected)***	**HS**	**PD↓↑**	***x***	***y***	***z***	***T***	***p (uncorrected)***
**BASAL GANGLIA (VOI)**														
Cluster left striatum	+	↓	−12	16	−4	4.4	0.04							
Cluster thalamus/pulvinar									↑	14	−26	4	5.9	0.001
**CEREBELLUM**														
Anterior								+	=	18	−50	−22		
Posterior									↑	−32	−54	−32	6.5	*p* < 0.001
								+	↑	32	−64	−32	4.7	*p* < 0.001
**CEREBRAL CORTEX**														
SMA (BA6)	+	↓	8	−16	50	3.1	0.002							
Medial prefrontal (BA9)	+	↓	−12	34	44	4.1	*p* < 0.001							
Dorsolateral PMC (BA6)	+	↓	−34	−16	64	5.4	*p* < 0.001		↑	38	−66	44	4.3	*p* < 0.001
	+	↓	34	−12	64	3.7	*p* < 0.001							
M1 (BA4)	+	↓	−38	−22	52	3.9	*p* < 0.001							
Mid Temporal (BA39)	+	↓	−54	−64	24	3.6	0.001							
									↑	40	−60	24	5.0	*p* < 0.001
Superior parietal (BA7)	+	↓	18	−60	58	2.8	0.004		↑	34	−64	54	4.7	*p* < 0.001
V1 (BA17/18)	+	↓	−10	−84	0	4.7	*p* < 0.001							

*Brain activations for comparisons between singular and composite movement and between healthy subjects (HS) and Parkinson's disease patients (PD). “+,” sign indicates activity in the masked condition for healthy subjects; “↑↓,” signs indicate increased or decreased activations in PD patients compared to HS. Co-ordinates refer to the voxels of maximum activation within significant clusters (voxel level, uncorrected). Positive x, y, z coordinates (in mm) indicate locations right, anterior and superior of the middle of the anterior commissure, respectively*.

#### Between-group comparisons: PD patients vs. healthy subjects

**Figure 5:**
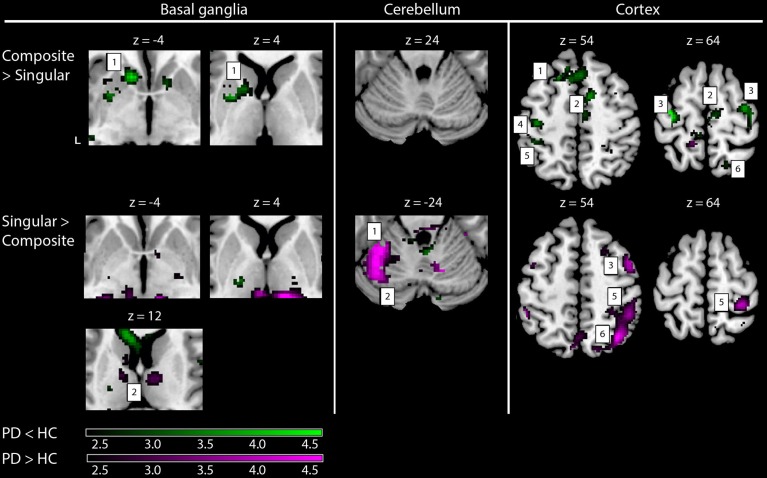
**fMRI results: differences between healthy subjects and Parkinson patients**. Increased activations (SPM T-maps) for the two comparisons between composite and singular step-track movements. Green activations: PD < HC, purple activations: PD > HC. Basal ganglia: 1, left striatal cluster; 2, thalamic/pulvinar cluster. Cerebellum: 1, anterior cerebellum; 2, posterior cerebellum; Cortex: 1, medial prefrontal (BA9); 2, supplementary motor area (BA6); 3, (dorsal) premotor cortex; 4, primary motor cortex (BA4); 5, primary sensory cortex (BA2); 6, superior parietal cortex (BA7). Differences in activations between groups were investigated by using exclusive masks (at threshold level *p* = 0.05). For visualization purposes, all activations are shown above a threshold level of *p* = 0.01 (uncorrected), without a restriction of cluster size (k). The z-coordinates indicate the position of the shown transversal planes relative to the AC–PC plane. Activations were rendered on the standard anatomical (ch2) template of MRICron (Rorden et al., 2007). L, Left hemisphere.

Comparing the patterns of brain activations between groups using exclusive masking, revealed that for composite movements (Composite > Singular) patients showed decreased activations in the left ventral striatum (Figure 5 and Table 4). In patients compared to healthy subjects, decreased cortical activation was also found in the SMA and bilateral (pre-) motor areas, the contralateral medial prefrontal and ipsilateral superior parietal cortex, while increased activation was seen mid temporally. For the comparison Singular > Composite, patients had decreased activation in the contralateral ventro-lateral thalamus. They showed increased activations in a cluster of the right pulvinar, extending to the bilateral anterior thalamus and dorsal caudate. The comparison Singular > Composite showed increased activations in patients distributed over the ipsilateral dorsal PMC, superior parietal and contralateral posterior cerebellum (lobule VI).

## Discussion

The employed step-tracking task, requiring subjects to make similar movement excursions into various directions, enabled a balanced dissociation between composite and singular movements. By investigating this dissociation in directional movements at behavioral and cerebral level, both in healthy subjects and PD patients, we were able to demonstrate a relation between composite movements and putative cortico-striatum circuitry, whereas cortico-cerebellar circuitry was stronger implicated in singular movements.

The fMRI results showed that decreased striatal activation was related to impairment of composite movement in PD, while singular movement in patients was related with increased right parietal and left cerebellar activation when compared to healthy subjects. The association between these brain, behavioral and muscle activity findings suggests that PD-related changes in cortico-striato-cortical function result in an impaired ability to select synergistic patterns of motion that demand particularly highly tuned muscle activity.

We acknowledge that the fMRI results were only identified at lenient statistical thresholds. This limitation of the study might raise valid critique if the identified clusters would have been without logical functional anatomical coherence. The fact that the patterns of activation did represent such coherence, both between striatum and ipsilateral cortical effects and between cerebellum and contralateral cortex activations, made us confident that these results represented physiological effects.

### Healthy subjects characteristics

#### Behavioral level

The a priori dichotomous classification of movement directions was confirmed by the two patterns of muscle activities. While the different movement directions shared common features such as movement amplitude, timing and speed, we found that composite movements did indeed involve more muscles than singular movements, as indicated by the muscle activity index (AI). The distinction between direction-associated singular and composite movements suggests a distinction between efficient and less efficient movement directions, which may particularly be due to anatomical characteristics such as the possible movement excursions in the wrist and position of muscle insertions, while gravity may be an additional external factor. In the end, the brain accomplished to reach similar movement results, given the described similarities in movement amplitude and speed.

Regarding the effect of gravity, movements in the directions 45°, 90°, and 135° require higher agonist activity to overcome gravitational effects and lower antagonist activity to terminate the movement, vs. less agonist activity and more antagonist activity for movements in the opposite, more downwards directions 225°, 270° and 335°, when the movement is “assisted” by gravity. Such physical characteristics are invariant parameters the brain has to deal with when organizing purposeful movement. Apparently, finding an optimal way of coupling various muscles contributes to solving these constraints. Regardless the cause of the dichotomy between singular and composite movements, these differences in muscle co-activity tuning provided specific parameters to investigate the underlying cerebral organization.

#### Cerebral level

The present fMRI results indicate that composite movement in healthy subjects is characterized by **left striatal activity**, corroborating the important role of the BG in selection of appropriate movement (Mink, 1996, 2003; Grillner et al., 2005; Lehericy et al., 2006). Furthermore, the co-occurrence of SMA activation is consistent with its role in movement selection (Deiber et al., 1999; Neubert et al., 2010). On the other hand, while *composite* movement elicits activation of the BG as well as cortical sensorimotor and premotor areas, *singular* movement was characterized by activation of the (contralateral) ventro-lateral thalamus and ipsilateral cerebellum. This combination of activations is in accordance with the well-described functional connection between the contralateral thalamus and ipsilateral cerebellum in monkeys (Asanuma et al., 1983; Sakai et al., 1996). Higher activation of particularly the anterior lobe of the cerebellum found in the present study may emphasize its “corrective” role in movement optimization (Glickstein, 1992; Wolpert et al., 1998; Spencer et al., 2005). Such “corrective” aspects may become particularly urgent when movements are controlled by only a few opposite muscles. The latter may easily result in oversized movement excursions. The effective result of this putative cerebellar contribution is supported by less variability in movement execution at the behavioral level. Thus, we found a dissociation between BG involvement in highly tuned muscle co-activity, requiring more extensive planning and preparation to obtain adequately tuned patterns of co-active muscles, and cerebellar activation during movements requiring less muscle co-activity but more direct agonist-antagonist corrections. This may reflect the complementary roles of these areas in motor control in which the BG are involved in movement selection, whereas the cerebellum has a role in movement optimization (Stein and Glickstein, 1992; Jueptner and Weiller, 1998; van Donkelaar et al., 2000; Bostan et al., 2010).

### PD patients in comparison to healthy subjects

#### Behavioral level

As hypothesized, patients showed less specialized muscle activity patterns than healthy subjects. Although directions requiring maximal muscle activity were the same in patients and healthy subjects (Figure 3), patients showed more muscle co-activity in the remaining directions, resulting in a less differentiated pattern and lower DQ. Similarly, patients employed more muscles for movements than healthy subjects, as indicated by a higher AI, particularly in singular, but also in composite movements. These findings imply decreased capacity to select appropriate muscle synergies. In addition, patients showed higher RTs and higher RT variability regardless of direction, which is in accordance with other studies investigating movement performance in PD (Majsak et al., 2008; Dounskaia et al., 2009). Moreover, the kinematic parameters RT variability and mean PV indicated a decline in motor performance in PD patients particularly for composite movements. Thus, PD-related changes in motor performance were most evident for composite movement and indicate that PD patients are especially impaired on tasks requiring highly tuned muscle co-activity.

#### Cerebral level

At cerebral level, the PD-related decreases in activation within the contralateral striatum and interconnected circuitry during movements with highly tuned coordination of co-active muscles are in accordance with the classic PD model, although we had expected to find more extensive decreases in activation in the BG. The PD model describes a striatal dysfunction that induces enhanced inhibitory BG outflow to the thalamus and subsequently to the cortex (Albin et al., 1989; DeLong, 1990; Boecker et al., 2008; Obeso et al., 2008). The association between our fMRI results and decline in motor performance further underlines the role of the BG in movement selection. As compared to healthy subjects, the cortical increases in activation in PD during singular movement were particularly evident in the ipsilateral cortex and included the PMC, sensorimotor and parietal cortex. This ipsilateral distribution suggests the involvement of higher-order aspects of motor control. One may, in this respect, consider a stronger reliance on visual information in PD, through the parietal-premotor network (Praamstra et al., 1998; de Jong et al., 1999). Moreover, the ipsilateral (right) parietal cortex was found to be prominently active. This is in accordance with its involvement in visual processing and control of spatial attention (Gottlieb and Snyder, 2010) which is considered to be right hemisphere dominant (Malhotra et al., 2009; Thakral and Slotnick, 2009). By controlling shifts of spatial attention, as required during a task with shifting visual cues such as step-tracking, the parietal cortex plays a role in action selection (Cisek, 2007). By modulating selection via the PMC, the parietal cortex influences motor processing; an effect that seems to be stronger in patients as compared to healthy subjects during singular movement. Additionally, we found that patients had increased activation in the superior posterior lobe of the left cerebellum. A contribution of this cerebellar region to visuospatial processing has been previously described (Stoodley and Schmahmann, 2009) and is consistent with distinct impairments on spatial tasks after damage of the left cerebellum (Gottwald et al., 2004; Hokkanen et al., 2006). Furthermore, a functional interaction between the posterior cerebellum and the opposite parietal cortex is effectuated by (crossed) connections (Sasaki et al., 1975). This interaction was further supported by a study on perception of hand movement that found a functional relation between the left posterolateral cerebellum and the right parietal cortex (Hagura et al., 2009).

These findings suggest a compensational mechanism involving the parietal cortex and the cerebellum. Compensational activation in PD patients involving the cerebellum is supported by the findings in the fMRI study of Yu et al. (2007), who examined differences in activation patterns during a simple, paced thumb pressing task and found significantly higher activations in the cerebellum in PD patients. These findings lead to the hypothesis of a compensational mechanism involving the cerebellum. In contrast to their study, our study was designed to differentiate between different movement tasks (requiring high- vs. low-tuned muscle activity, respectively). This allows us to extend the hypothesis of compensational cerebellar activation in PD patients to a hypothesis that this may indeed be task-specific. Therefore, we propose that the increased activation of the left posterior cerebellar lobe and right parietal cortex in PD patients is due to increased reliance on visuospatial processing, possibly as a compensational strategy in the context of impaired BG selection.

## Conclusion

In the present study, we demonstrated a dissociation between high- and low-tuned muscle activity patterns for various directions of center-out step track movements of the right hand. The latter could thus be characterized as singular and composite movements, which were each related with a specific patterns of brain activation. These two movement-related activation patterns showed differential changes in PD patients when compared to healthy subjects. In healthy subjects, we found a striking dissociation between involvement of the striatum and cortical areas in composite movement, vs. cerebellar involvement in singular movement; findings that may reflect the complementary roles of these areas in motor control. In patients we found decreased activation of the striatum and interconnected cortical areas for composite movement together with a decline in motor performance. These changes at both cerebral and behavioral level indicate that, as a result of changed cortico–striato–cortical functionality, PD patients are particularly impaired on tasks requiring highly tuned muscle co-activity. In singular movement, PD patients performed better and showed a combination of increased activation in the ipsilateral parietal cortex and left cerebellum. We interpret this as increased visuospatial processing, possibly deployed as a compensational mechanism.

## Author contributions

CT, BD, NM, BC, and KL conception and design of research; CT performed experiments; AV, CT, and PY analyzed the data; CT, AV, BD, and NM interpreted results; GV and BC contributed analysis tools; AV, CT, and PY drafted manuscript; AV, CT, BD, BC, NM, and KL edited and revised manuscript, CT, AV, BD, and NM approved final version of manuscript.

## Funding

This study was sponsored with a grant from the International Parkinson Foundation (IPF), grant title: “Initiation and Inhibition of Movement in patients with Parkinson's disease: New Insights Using a Direct Coupling between Movement, Muscle and Brain Activity.”

### Conflict of interest statement

The authors declare that the research was conducted in the absence of any commercial or financial relationships that could be construed as a potential conflict of interest.

## Abbreviations

ANOVA: analysis of variance
AI: activation index
BA: Brodmann area
BG: basal ganglia
DQ: differentiation quotient
EMG: electromyography
fMRI: functional magnetic resonance imaging
M1: primary motor cortex
m. ECRB: (musculus) extensor carpi radialis brevis
m. ECRL: (musculus) extensor carpi radialis longus
m. ECU: (musculus) extensor carpi ulnaris
m. FCR: (musculus) flexor carpi radialis
PD: Parkinson's disease
PMC: premotor cortex
PV: peak velocity
ROI: region of interest
RT: reaction time
SMA: supplementary motor area
UPDRS: Unified Parkinson's Disease Rating Scale.
